# Genetic trajectory and immune microenvironment of lung-specific oligometastatic colorectal cancer

**DOI:** 10.1038/s41419-020-2480-6

**Published:** 2020-04-24

**Authors:** Alessandro Ottaiano, Luisa Circelli, Angela Lombardi, Stefania Scala, Nicola Martucci, Jerome Galon, Manuela Buonanno, Giosuè Scognamiglio, Gerardo Botti, Fabienne Hermitte, Giovanni Savarese, Luigi D’Amore, Fabiana Tatangelo, Annabella Di Mauro, Giuseppina Liguori, Anna Maria Trotta, Maria Napolitano, Monica Capozzi, Salvatore Tafuto, Francesco Perri, Antonello La Rocca, Michele Caraglia, Guglielmo Nasti

**Affiliations:** 1 Department of Abdominal Oncology, SSD-Innovative Therapies for Abdominal Cancers, Istituto Nazionale Tumori di Napoli, IRCCS “G. Pascale”, , Via M. Semmola, 80131 Naples, Italy; 2 AMES-Centro Polidiagnostico Strumentale, Srl, Naples, Italy; 3 Department of Precision Medicine, University of Campania “L. Vanvitelli” , Via L. De Crecchio, 7, 80138 Naples, Italy; 4 Functional Genomics, Istituto Nazionale Tumori, IRCCS “G. Pascale” , Via M. Semmola, 80131 Naples, Italy; 5 Department of Thoracic Surgery and Oncology, Istituto Nazionale Tumori di Napoli, IRCCS “G. Pascale” , Via M. Semmola, 80131 Naples, Italy; 6 INSERM, Laboratory of Integrative Cancer Immunology, Equipe Labellisée Ligue Contre le Cancer, Sorbonne Université, Université Sorbonne Paris Cité, Université Paris Descartes, Université Paris Diderot, Centre de Recherche des Cordeliers, 75006 Paris, France; 7 Center for Radiological Research, Department of Radiation Oncology, Columbia University Medical Center, New York, New York, NY USA; 8 Department of Pathology, Istituto Nazionale Tumori di Napoli, IRCCS “G. Pascale”,, Via M. Semmola, 80131 Naples, Italy; 9 HalioDx, Marseille, France; 10 Department of Abdominal Oncology, Clinical and Experimental Abdominal Oncology, Istituto Nazionale Tumori di Napoli, IRCCS “G. Pascale”, Via M. Semmola, 80131 Naples, Italy; 11 Head and Neck Cancer Medical Oncology Unit, Istituto Nazionale Tumori di Napoli, IRCCS “G. Pascale”, Via M. Semmola, 80131 Naples, Italy; 12 Biogem Scarl, Institute of Genetic Research, Laboratory of Precision and Molecular Oncology, Ariano Irpino, Italy

**Keywords:** Predictive markers, Colorectal cancer

## Abstract

Genetics and immunologic dynamics pushing the evolution of colorectal cancer (CRC) from the primary tumor to the metastases are largely unknown; cancer heterogeneity makes challenging both therapy and mechanistic studies. We selected patients developing CRC with lung-limited metastatic disease as only illness during their life in order to find any relevant genotype–phenotype relationship. Analysis of 523 cancer-relevant genes and of immune cells infiltration in primary and metastatic tissues revealed atypical genomic trajectories (TMB decrease, *KRAS* and *SMAD4* regressive mutations), specific genetic events (*ERBB2* point mutations) and scarce T-cell infiltration. These insights provide novel information in oligometastatic CRC biology and new perspectives for cancer monitoring and anti-cancer therapeutic strategies.

## Introduction

**Fig. 1:**
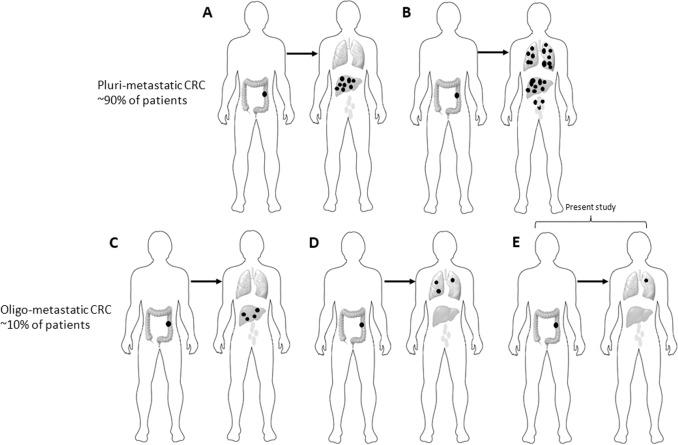
Some common clinical patterns of CRC metastasis formation. Pluri-metastatic disease with **a** wide diffusion to liver or **b** to multiple organs (most frequently including liver, lungs, lymphnodes), and oligometastatic disease with “low burden” single organ involvement (liver in **c**, lungs in **d**). The disease pattern here studied is depicted in **e**.

Colorectal cancer (CRC) is the third most frequent neoplasm and the second cause of cancer-related deaths worldwide^1,2^. In clinical practice, most of metastatic CRC patients present with disseminated nodules (pluri-metastatic disease, pMD) involving liver (about 40%) or more than one organ (about 50%) (Fig. 1a and b); the remaining mCRC patients (5–10%) present with indolent and low burden disease (liver and/or lung, ≤3 nodules per organ)^3^ (Fig. 1c–e). Identifying the genetic and immunologic events in determining the clinical behavior and the evolution of a neoplasm, in time and space, is a new frontier of cancer research since the introduction of high-throughput genetic analyses. Such analyses provide novel insights in cancer biology and are crucial to identify new therapeutic strategies.

Many studies in pMD of CRC and other cancers have revealed heterogeneous results in genetics of primary tumors (PT) and matched metastatic lesions (as evidenced by de novo variations) with concordance rates (shared point mutations/total number of point mutations) varying from 0 to 100% (median: 45%)^4–8^ (Supplementary File S1). The last evidences suggest that, when genetically similar, the cancer might evolve via epigenetic or regulatory modifications otherwise, crucial genetic events might switch on the metastatic/aggressive phenotypes (uncontrolled and sustained proliferation, spread to distant specific organs, resistance to therapy, etc.). Furthermore, according to the immunoediting hypothesis^9^, the large part of tumors, before developing immuno-evasive mutations, would be recognized and sculptured by lymphocytes. However, the timing as well as the clinical effects of such internal clash among altered neoplastic cells and host lymphocytes are extremely complex and still largely unknown.

Previous studies of evolution of CRC metastatic lesions as compared to the primary tumors suffer of the extreme heterogeneity in terms of patients, disease features, anti-cancer treatments, disease spread and sites of metastases. These factors could account for heterogeneous results and interfere with the cancer phenomenology study.

Thus, in the present study, an a priori strict enrolment was conducted to select mCRC patients in order to (i) minimize any clinical and environmental interferences and (ii) find any specific genotype–phenotype relationships. Our aim was to investigate the impact of cancer heterogeneity (through next-generation sequencing—NGS) and patients’ immunologic dynamics (through characterization of cancer immune microenvironment) on the development of lung-limited, single-nodule metastatic disease (Fig. 1e) in CRC. Such clean model can contribute to explore and generate hypotheses on the genetic trajectories and immunologic dynamics underlying oligometastatic CRC evolution and lung site-specific dissemination.

## Results

### Patients, disease characteristics and genetic concordance

**Fig. 2:**
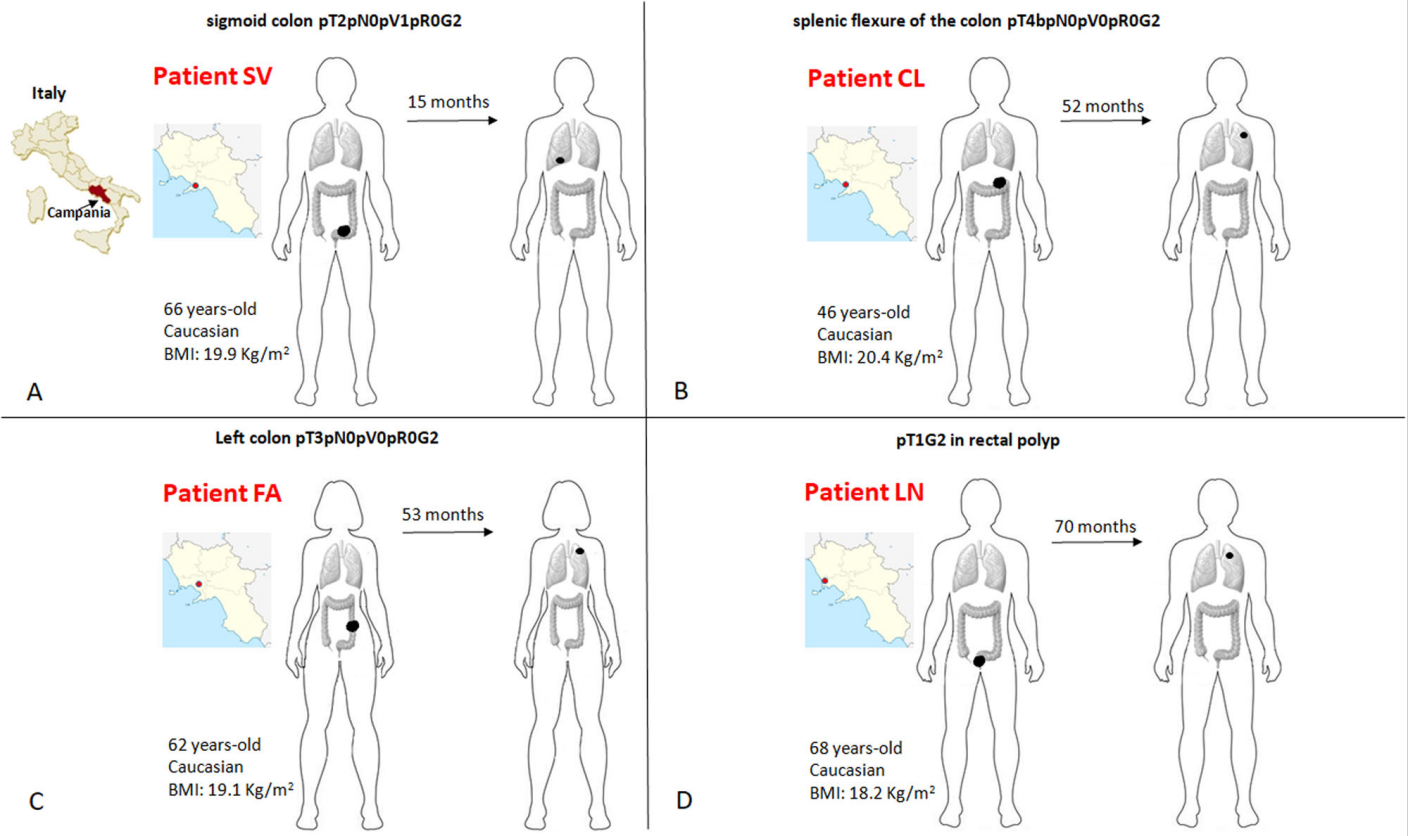
Detailed clinico-pathological characteristics of patients. Schematic representation (**a**–**d**) of patients’ geographical origin, stage at diagnosis, age, race, body mass index (BMI), localization of primary tumor, time elapsed from the initial diagnosis to the development of radiologically evident lung metastasis (above the *arrow*), localization of lung metastasis. The cases’ panel order reflects the time-to-lung metastases (from from the shortest to the longest time elapsed: SV 15 m, CL 52 m, FA 53 m, LN 70 m).

From 2006 to 2016, 97 patients underwent to lung wedge resections for CRC oligometastases (1–3 nodules). As reported above, a strict selection of those patients was subsequently applied in order to identify CRC patients who developed a single metastasis and were free from disease recurrence at a minimum follow-up of three years after lung resection. In addition, patients who had received long-lasting adjuvant chemotherapy (more than four cycles of fluoropirimidines and oxaliplatin) or presented with any chronic illness (diabetes, hypertension or other cardiovascular diseases, chronic infections, auto-immune or inflammatory diseases, other cancers) were also excluded. The complete inclusion and exclusion criteria with the relative flow-chart are showed in (Supplementary File S2). Such strict selection should minimize potential interferences to explore the genotype–phenotype evidence relevant for the evolution from primary to single-nodule metastatic disease in CRC. Four CRC patients were selected (SV, CL, FA, LN). The cases’ plotting order in figures and tables reflects the time-to-lung metastases (from the shortest to the longest time elapsed: SV 15 m, CL 52 m, FA 53 m, LN 70 m). Sites, initials, genders, ages at diagnosis, body mass indexes, pathologic stages, localizations of PT and MT (metastatic tumor), dates of diagnosis and lung recurrence are shown in Fig. 2. Patients and tumor characteristics according to tumor mutation burden (TMB) and genetic sharing between PTs and MTs are shown in Table 1. TMB decreased from PT to MT except for CL in which TMB increased (CL case: 7.0 mut/Mb PT vs 694.3 mut/Mb MT). The genetic sharing is high in SV (81.9%) and LN (95.9%), low in CL (12.2%) and FA (13.3%). Furthermore, to describe the matched mutational profiles of primary and metastatic lesions, mutational signatures (Methods) were depicted in (Supplementary File S3). Interestingly, patients CL and FA had also higher PT/MT differences in terms of mutational profiles [>80% of the 96 combinations with a Δfrequencies (MT-PT) of specific base-pair mutation types >25%] compared to SV and LN.

**Table 1:** Clinicopathological characteristics and genetic concordance in the enrolled patients.

Patient Initials	PT localization	Pathologic stage at diagnosis	Max diameter of PT at gross dissection (cm)	Adjuvant chemotherapy	IS in PT	Time-to-lung metastasis (months)	Max diameter of nodule at CT scan (cm)	TMB in PT (mut/Mb)	TMB in MT (mut/Mb)	No. of shared mutations	Genetic sharing PT/MT	Number of new coding genetic variants in MT
SV	Sigmoid colon	pT2pN0pV1pR0G2	4.0	No	Low	15	1.2	1.9	1.6	141	81.9%	31
CL	Left colon	pT4bpN0pV0pR0G2	3.5	Four cycles (CAPOX)	Int	52	1.3	7.0	694.3	25	12.2%	40
FA	Left colon	pT3pN0pV0pR0G2	3.0	Two cycles (CAPOX)	Int	53	2.0	21.0	6.3	24	13.3%	149
LN	Rectum	pT1pNpV0pR0G2	2.5	No	NE	70	1.1	8.8	8.6	165	95.9%	3

*CAPOX* capecitabine and oxaliplatin, *CT* computed tomography, *Int* intermediate, *IS* ImmunoScore, *MT* metastatic tumor, *NE* not evaluable, *PT* primary tumor; *TMB* tumor mutation burden.

### Mutations’ evolution and MSI testing

**Fig. 3:**
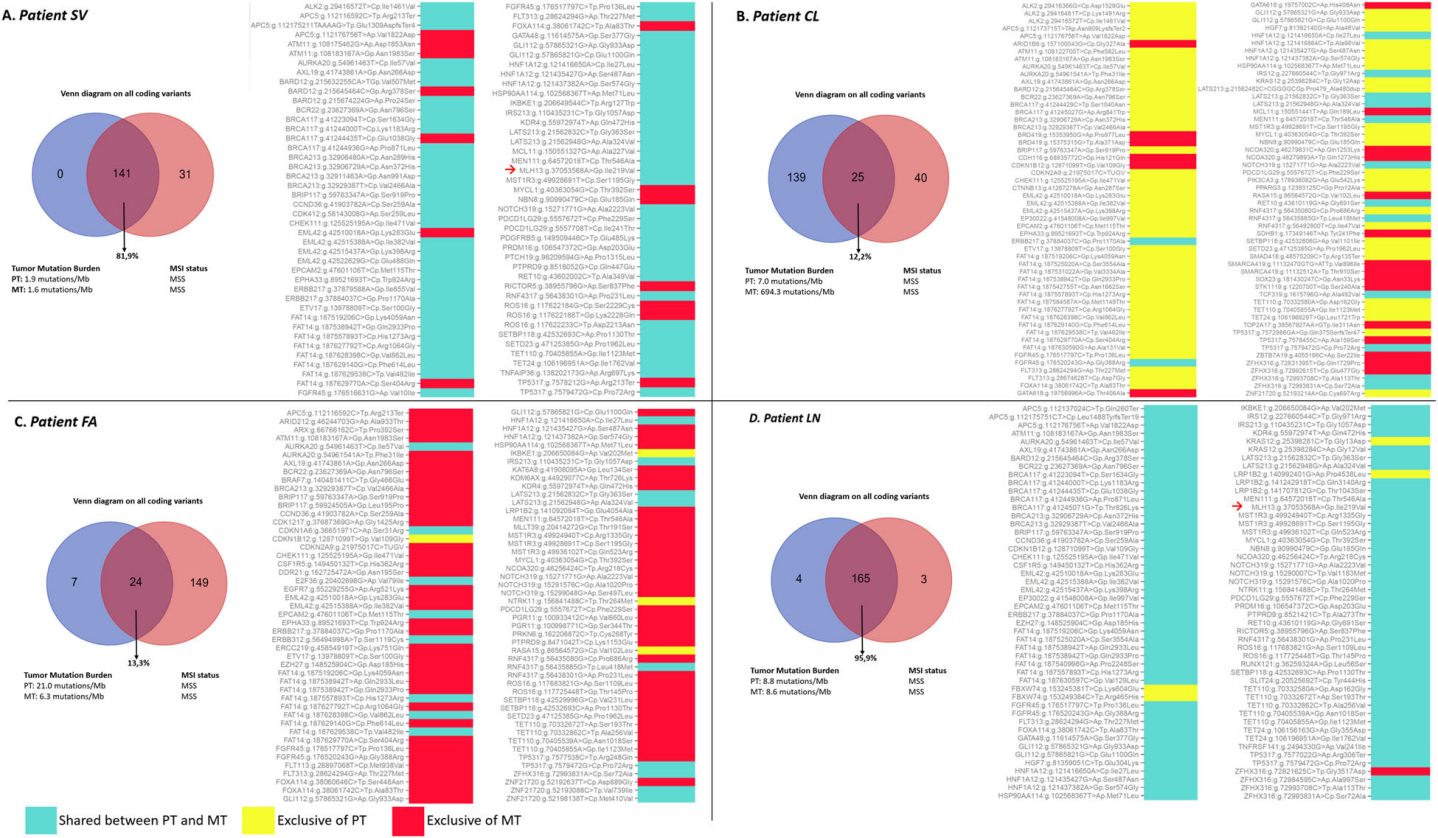
Primary and metastatic tumor genes comparison. Venn Diagrams, tumor mutation burden (TMB), microsatellite instability (MSI) status (*left panel*) and descriptive pairwise comparison heatmap of all coding genetic variants of strong or potential/unknown clinical significance (*right panel*) for each patient (**a** SV, **b** CL, **c** FA, **d** LN). Cyan indicates shared genetic variations, yellow PT private variations, red MT private variations. Coding variations in MSI-associated genes of potential clinical significance are indicated, if present, with a red arrow. “Variant calling” and “TMB calculation” were based on not related algorithms, thus the real number of coding variants cannot be derived from TMB and vice versa (see Manufacturer Instructions at https://emea.support.illumina.com/).

The genetic tumor evolution from PT to MT is shown in (Fig. 3a–d) according to strong/potential/unknown (Tier1-3) AMP/ACMG prioritization of variants. The genetic sharing PT/MT is also depicted in Venn Diagrams embedded into Fig. 3; the TMB ranged from 1.6 mutations/Mb (SV metastasis) to 694.3 mutations/Ml (CL metastasis). In all cases except one (CL), there was a reduction in TMB from PT to MT. All cases were MSS in both PT and MT. MSI-associated genes of potential clinical significance are indicated with a red arrow; only SV and LN MLH variant was common (p.Ile219Val).

**Fig. 4:**
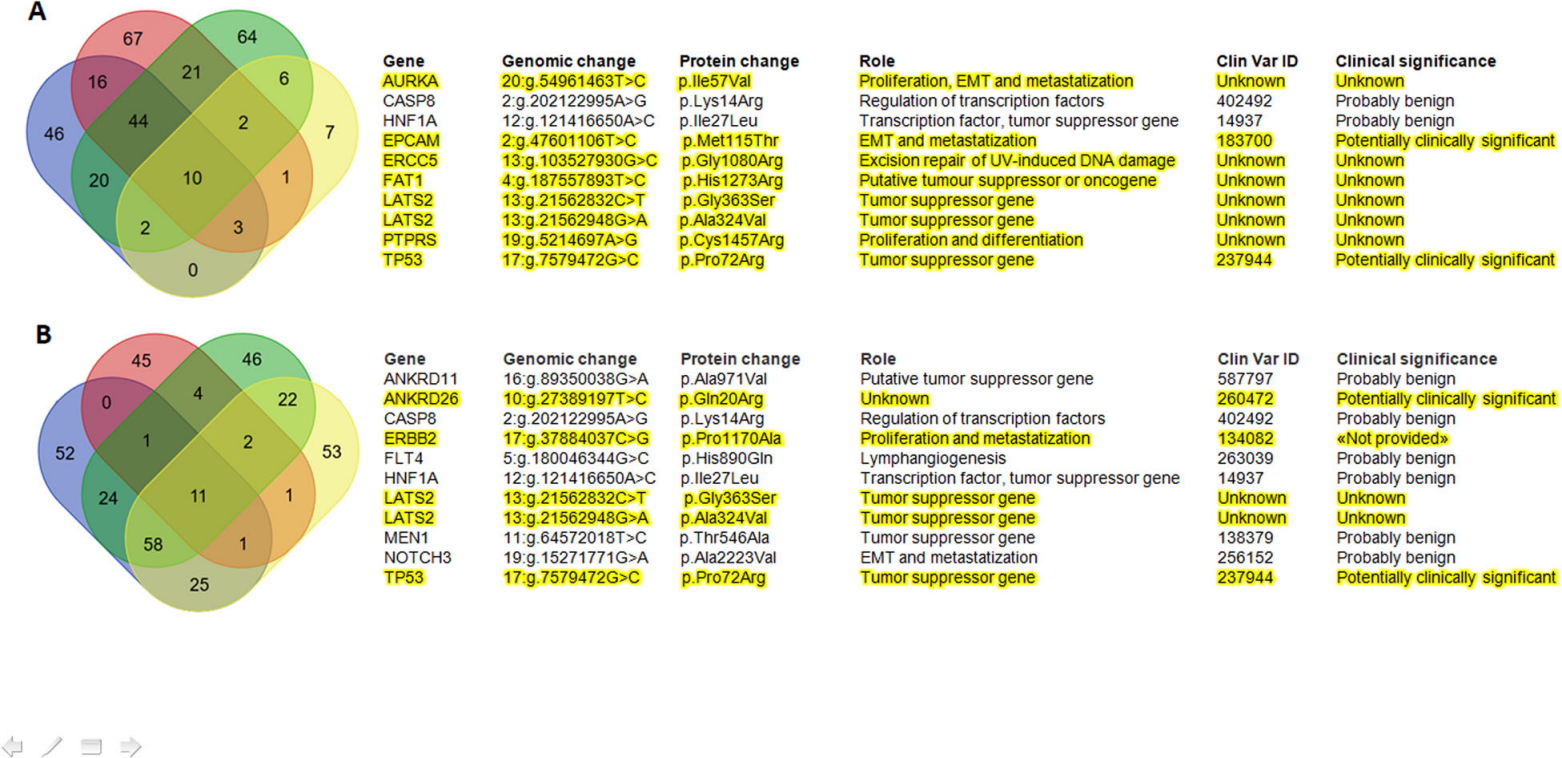
Tumors’ genetic sharing. Venn Diagrams on variants shared by primary tumors (**a**) and metastatic tumors (**b**). Benign variants are not highlighted and are excluded from pattern A and pattern B definition (see also Results section). Variants are indicated with gene name, genomic and protein change, their role in cancer, their ClinVar ID with clinical significance interpretation (see also Methods section).

**Fig. 5:**
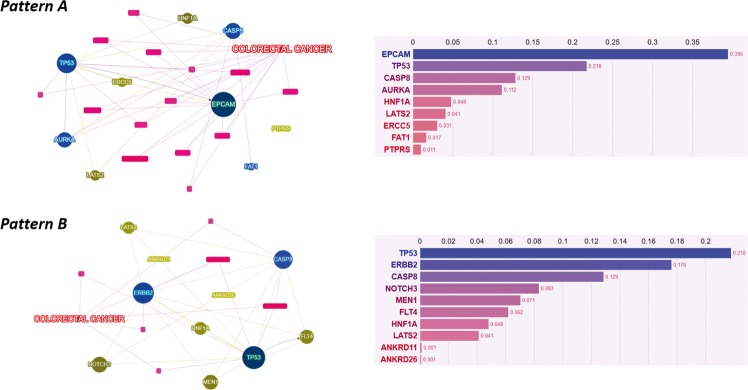
Genetic variants prioritization. Phenolyzer genetic variants prioritization according to **a** pattern A (previously defined in primary tumors) and **b** pattern B (previously defined in metastatic tumors).

The spectrum of CRC “driver mutations”, in a case-matched manner, is reported in Table 2. NGS allows the identification of hundreds of potentially significant mutations in cancer tissue specimens. Despite tumor heterogeneity, it is disputed and hypothesized that some backbone mutations might represent key events in determining cancer-specific phenotypes. Moreover, mutational concordance represents an indirect evidence that those mutations may have an important role in an evolution trajectory and/or in the establishment of metastases. Therefore, in order to identify genetic common patterns in both PT and MT, Venn diagrams of shared mutations have been depicted for both PTs (Fig. 4a, pattern A) and MTs (Fig. 4b, pattern B). The identified genes are described with ClinVar dataset ID and their role in cancer. Interestingly, some mutations of unknown or potential clinical significance recurred in all PTs (*AURKA, EPCAM, ERCC5, FAT1, LATS2, PTPRS, TP53*) and MTs (*ANKRD26, ERBB2, LATS2, TP53*). The genetic alterations shared (pattern A and B) in our series were different from that previously reported in pluri-metastatic CRC (**see** Supplementary File S1). In particular, *SMAD4* was present only in PT of CL case, *APC* was not present in PT of FA and MT of CL. Most surprisingly, *KRAS* mutations were not concordant in two cases. CL lost *KRAS* mutation in MT and LN lost a pathogenic mutation of *KRAS* in MT. *ERBB2 p.Pro1170Ala* was present in all cases except for PT of FA. Furthermore, we submitted our genetic results to Phenolyzer to depict relevance and relationships between any “seed” genetic variants and “secondary” ones (Methods). In PTs *EPCAM, TP53, CASP8* were the most dominant and interrelated genes, in MTs *TP53, ERBB2* and *CASP8* (Fig. 5).

**Table 2:** Summary of patient by patient CRC key driver mutated genes (strong or potential clinical significance according to four-tiered structure of AMP/ACMG consensus).

Patient	PT mutated genes	MT mutated genes
SV	APC, ERBB2, TP53	APC, ERBB2, TP53
CL	APC, ERBB2, KRAS, PI3KCA, SMAD4, TP53	ERBB2, TP53
FA	RASA1, TP53.	APC, BRAF, ERBB2, TP53
LN	APC, ERBB2, KRAS (two pathogenic mutations KRASp.Gly12Val and KRASp.Cly13Asp), TP53	APC, ERBB2, KRAS (p.Gly12Val), TP53

*MT* metastatic tumor, *PT* primary tumor.

### Tumor microenvironment characteristics

**Fig. 6:**
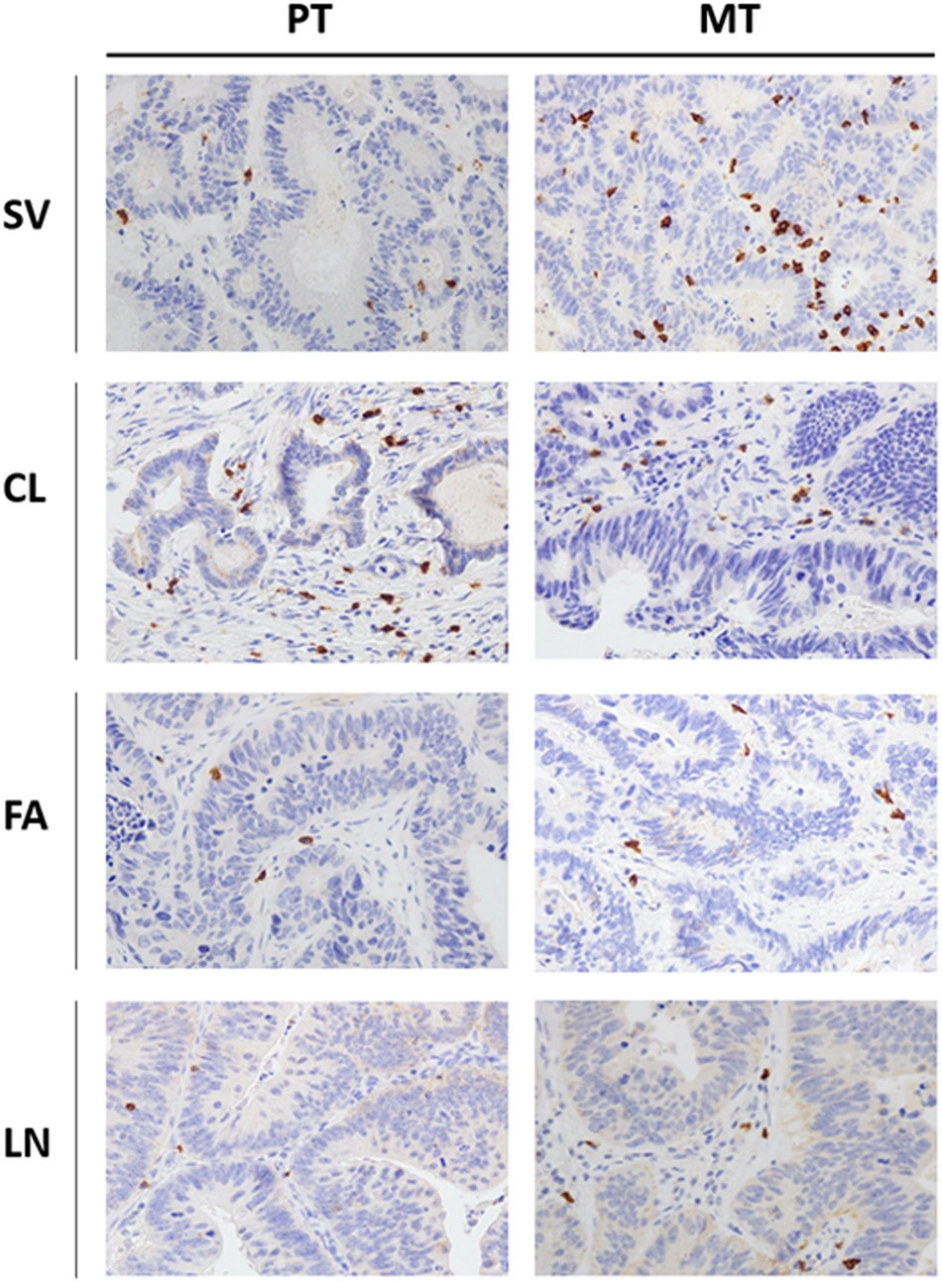
Tumor microenvironment immune context. Representative immunohistochemistry of CD8^+^ cells infiltrating primary (PT, *left panels*) and matched lung metastatic tumors (MT, *right panels*) (magnification: ×40 HPF—high-power field) for each patient (SV, CL, FA, LN).

**Fig. 7:**
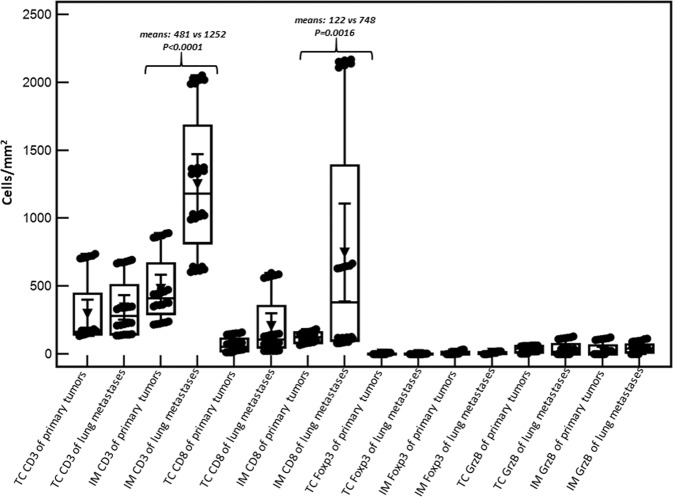
Box-and-wisher plots reporting lymphocytes densities (cells/mm^2^) in tumor cores (TC) and invasive margins (IM) of pooled primary and metastatic tumors. Significant differences (see Methods) are reported in the graph area.

Since the adaptive immune context of CRC has been associated with prognosis, CD3^+^, CD8^+^, FoxP3+ and GrzB+ cells were quantified in matched PT and MT tissues by IHC. The density of each T-cell subset is shown in Table 3. Notably, the density means of CD8^+^ and GrzB^+^ cells (the principal anti-tumor effectors) recorded in PTs (68 and 36 cells/mm^2^, respectively) were below the upper limit of the lowest quartile reported in a previous referral study^10^ (235 and 100 cells/mm^2^, respectively) as well as in our internal validation datasets. Results of total CD3^+^ and FoxP3^+^ cells are also reported in Table 3 and compatible with the previous report. Example of CD8^+^ T-cell subsets IHC staining (high-power field ×40) in PTs and matched MTs is reported in Fig. 6. In summary, our results point out a scarce T-cell effectors infiltration into PT samples. An external validation, blinded to clinical information and to our IHC results, was carried out by performing Immunoscores^11^ on three out four PTs (LN failed because of technical issues). To date, Immunoscore is the most objective (through digital pathology and a dedicated image-analysis software) and complex evaluation of the intra-tumoral immune context; it integrates information about types, density and location of tumor-infiltrating lymphocytes. Results of Immunoscore analysis are also shown in Table 1. The assessed cases did not present high Immunoscore consistently with our IHC analyses. Density of T-cell subsets in metastases were much more variable and were reported in Table 3 and depicted in Fig. 7. A phenotypic and functional characterization of peripheral lymphocytes is also reported in (Supplementary Files S4–8).

**Table 3:** Distribution of T-cell subsets densities (cells/mm^2^) in primary and metastatic tumors.

	Primary tumors								Metastatic tumors							
	CD3^+^		CD8^+^		Foxp3		GrzB		CD3^+^		CD8^+^		Foxp3		GrzB	
Patients	TC	IM	TC	IM	TC	IM	TC	IM	TC	IM	TC	IM	TC	IM	TC	IM
SV	170 ± 12.0	460 ± 20.5	80 ± 5.5	170 ± 12.5	10 ± 1.4	30 ± 4.2	60 ± 3.7	20 ± 3.1	340 ± 10.2	1350 ± 25.6	580 ± 20.3	2140 ± 30.9	0 ± 0.0	20 ± 1.6	35 ± 3.5	50 ± 1.1
CL	140 ± 7.0	875 ± 17.5	150 ± 10.6	152 ± 12.2	0 ± 0.0	5 ± 0.9	20 ± 0.8	0 ± 0.0	680 ± 15.3	2020 ± 32.4	80 ± 5.8	650 ± 18.7	0 ± 0.0	0 ± 0.3	0 ± 0.4	30 ± 3.2
FA	160 ± 9.5	365 ± 13.1	15 ± 1.2	75 ± 8.4	0 ± 0.0	0 ± 0.0	60 ± 5.1	115 ± 9.6	220 ± 9.9	1015 ± 23.8	140 ± 10.2	120 ± 8.7	5 ± 0.7	10 ± 1.3	0 ± 0.0	0 ± 0.0
LN	720 ± 16.5	230 ± 10.0	30 ± 2.4	95 ± 11.5	0 ± 0.0	0 ± 0.8	5 ± 0.6	0 ± 1.2	140 ± 7.2	625 ± 20.1	25 ± 2.2	85 ± 5.5	0 ± 0.0	5 ± 0.6	120 ± 11.2	100 ± 9.8

*Foxp3* forkhead box P3, *GrzB* Granzyme B, *IM* invasive margins, *TC* tumor core.

## Discussion

The evolutionary dynamics of cancer are highly debated and still largely unknown. It is not clear which are “timings” and “triggers” pushing the evolution of cancer from a single localized mass to a diffuse distant progeny. Our knowledge is very limited and only recently we started to approach the basic research with new technologies and methodologies. To this regard, the extended genomic characterization of primary and paired metastatic lesions from the same individuals represents the most meaningful approach to identify drivers genes or mechanisms involved in tumor progression. In vitro assays and/or animal models, although useful in an exploratory and “hypothesis-generating” sense, are too far from the human mechanistic physiology. Some studies with “high-throughput” DNA sequencing techniques have already compared primary CRC tumors with paired metastases particularly in pluri-metastatic disease^4–8^; they are heterogeneous in terms of involved organs, treatments and patients’ characteristics. We have used those data as genomic referral patterns of pluri-metastatic CRC and to compare them with our results (Supplementary Table S1).

Comparing our genetic patterns to the pluri-metastatic disease we focused on KRAS and SMAD4. Interestingly, in all studies of pluri-metastatic disease the *RAS* mutation status of PT is maintained in the matched MT; by converse, in some cases *KRAS* mutations are exclusive events of metastases. In two cases of the present report, this condition is not satisfied (MT of CL lost *KRAS* mutation, MT of FA lost a *KRAS* pathogenic mutation); the scarce immune cell infiltrate would suggest that this phenomenon is more likely related to “back mutations” rather than to immune-mediated sub-clonal selection. These data were repeated and confirmed with PEPP re-sampling and *KRAS* mutation PCR-based testing (data not shown). Interestingly, we have recently reported that the inflammatory status in *KRAS* mutated mCRC patients does not predict the response to TSPP (thymidylate synthase poly-epitope-peptide) vaccine and, in our series, 2 out of 4 PTs had *KRAS* mutations and scarce immune cell infiltrate^12^.

*SMAD4* is a downstream effector of the transforming growth factor (TGF)-β signaling pathway which has a critical role in CRC progression (angiogenesis and EMT). SMAD proteins (2 and 3) are phosphorylated by activated TGF-β receptors, and thus bind SMAD4. The resulting complex translocate into the nucleus where it acts predominantly as a tumor-suppressor gene of TGF-β-related genes. *SMAD4* has been demonstrated, in advanced CRC, as an independent prognostic factor for both reduced disease-free and overall survival^13–15^. In our cases, we found mutation of *SMAD4* only in PT of CL. This is another evidence of “back mutation”. Interestingly, it was also reported that CCL15 secreted from SMAD4-deficient CRC cells recruited CCR1^+^ cells, promoting their metastatic activities to the lung. IHC analysis of lung metastases from CRC patients revealed that CCL15 expression was significantly correlated with loss of *SMAD4*, and that CCL15-positive metastases recruited ~1.9 times more numbers of CCR1^+^ cells than CCL15-negative metastases. Immunofluorescent staining showed that most CCR1^+^ cells around lung metastases were tumor-associated neutrophils, although a minor fraction was granulocytic myeloid-derived suppressor cells^16^. Future studies are needed to explore in deep the role of SMAD4 genetic variations in both oligometastases and lung-specific metastatic homing.

Venn diagrams and Phenolyzer tools were used to select and depict both genetic changes and their relevance. The most shared and relevant genetic changes occurred in EpCAM (Epithelial cell adhesion molecule), TP53 and caspase-8 in primary tumors and in TP53, ERBB2 and caspase-8 in metastatic ones. Interestingly, EpCAM and caspase-8 have been also involved in regulating proliferation, migration and adhesion to lung tissue; their alteration could be, at least in part, responsible for the homing towards lungs^17–20^. Another observation that arises from our data is that ERBB2 was frequently mutated (3 PTs, all MTs: p.Pro1170Ala). As a non-synonymous coding variant, this Ala variant of the *ERBB2* may alter the spatial conformation of the tail region and may affect tyrosine kinase activity^21,22^. *ERBB2* is a member of the ERBB family of membrane tyrosine kinase receptors. Other members are the epidermal growth factor receptor (EGFR), erbB-3 (which lacks kinase domain), and erbB-4. Notably, no ligands for ERBB2 have yet been identified but it can hetero-dimerize with any of the other three ERBB family receptors upon ligand binding. Hetero-dimerization activates autophosphorylation of the cytoplasmic tyrosine residues, which are able to bind a variety of signaling molecules involved in proliferation, migration and angiogenesis. Evidence have been accumulated on the role of ERBB2 amplification in cancer, while very little is known on the role of point mutations^23^. It was recently reported that patients with ERBB2-negative breast cancer with the Pro1170Ala polymorphism variant exhibit a decreased survival outcome^24^. Another study suggested that the Ala variant increased the risk of lung cancer, indicating that it may promote ERBB2 activity^25^. However, Minn et al.^26^ and Guttlein et al.^27^ showed that, in ERBB2 overexpressing breast cancer cell lines, lung colonization is predominant and mediated by SPARC (secreted protein acidic and rich in cysteine).

Although a quite convergent genotypic evolution was observed in case SN and LN, in cases CL and FA, there were much more private events in their metastatic lesions, with a genetic concordance of 12.2% and 13.3%, respectively. A similar result was obtained with mutational profiles. The latter (Methods) can be used as a powerful approach to depict the molecular similarity between PTs and MTs, representing the mutational underlying mechanisms specific for each tumor. Patients CL and FA had also high PT/MT differences in mutational profiles. Interestingly, these patients underwent adjuvant chemotherapy with capecitabine and oxaliplatin (FA two cycles, CL four cycles) (Table 1) before surgical resection of lung metastases. CL, who is the unique patient with increasing TMB (from 7.0 to 694.3 mut/Mb) was the patient who received more cycles of chemotherapy. The intriguing hypothesis supported by these data is that the treatment would concur to the heterogeneity of subsequent progressing cancer cells.

In the present study, we did a great methodological effort to minimize any external/internal factors interfering with the dynamics under study. Our challenge was to explore what happens from a genetic point of view in patients who developed CRC and a single metastatic lesion to lung. There are no data on such genotype/phenotype correlation in literature. We identified a quite homogeneous oligometastatic model: did it represent the extreme of the metastatic CRC “spectrum” or a specific disease? Surprisingly, our results were consistent with some of the rules which govern the evolution of species^28–31^. In fact, previous studies demonstrated that metastatic tumors derive from individual clones of heterogeneous primary tumors and follow the rules of Darwinian evolution^30,31^. Although our model was highly clean and very selected, we cannot establish, due to the small sample size, neither that chemotherapy induces genetic remodeling or back mutations (i.e. patient CL) during cancer evolution nor that these phenomena occurred spontaneously (i.e. patient LN). Similarly, we cannot rule out the hypothesis that the common scarce immune cell infiltration could be the consequence of other unknown underlying molecular alterations. Additional studies are needed to understand the forces regulating such genetic/immunologic trajectories. However, here, we suggest that in the oligometastatic phenotype the tumor clones might follow genetic trajectories (i.e. back mutations in specific genes) which differ from those of clones with polymetastatic properties (Supplementary Table S1). The identification of these differences can be pivotal in identifying prognostic/predictive markers as well as in planning revolutionary therapeutic strategies. Furthermore, while the “effect” of back mutated KRAS in reducing spread/aggressiveness of metastases is expected and reasonable, at this stage, we cannot exclude any compensatory unrevealed alteration justifying the oligometastatic behavior in the other patients without back mutations. It has also to be considered that the low Immunoscore of 3 out of 4 PTs and the scarce immunological infiltrate in all PTs suggest the development of a PT with poor immunogenicity and low propensity to diffusely metastasize but with some affinity for lung colonization.

Therefore, “back mutations” of driver genes and absence of an evolutionary pushing (i.e. immune system recognition) could be responsible of “tuning down” the evolutionary trajectory (aggressiveness/progression) of the cancer (Supplementary File S9). Future studies are urgently needed to explore the potential clinical applications of this small albeit intriguing piece to cancer evolution mosaic that here we provide.

## Subjects and methods

### Tumor specimens and sequencing

Matched formalin-fixed and paraffin-embedded (FFPE) tissue specimens of primary CRC and single-nodule lung metastases were collected from selected patients. The study was approved by the Scientific Directorate of our Institution (Prof. Gerardo Botti). Written informed consent was obtained from patients before starting the translational studies on peripheral blood and FFPE tissues. A complete list of inclusion and exclusion selection criteria is reported in Supplementary File S2. 10 mM-serial sections were cut from each tissue specimen for microdissection of tumor cells under morphological control. DNA isolation was performed through the MGF03-Genomic DNA FFPE One-Step Kit, according to the manufacturer’s protocol (MagCore Diatech). DNA quality was established in triplicate using the FFPE QC Kit according to the manifacturer’s protocol (Illumina, San Diego, USA). Libraries were prepared with TruSigtTMOncology 500 kit, based on target enrichment that analyzes 523 cancer-relevant genes (the list is reported in Supplementary File S10). The assay detects small nucleotide variants (SNVs), indels, splice variants and immunotherapy biomarkers such as tumor mutational burden (TMB) and microsatellite instability (MSI) (see below). Sequencing was performed on an Illumina NovaSeq 6000 (San Diego, USA) platform.

### Tumor mutational burden (TMB), microsatellite instability (MSI) and mutational profiles

TMB was measured by exome sequencing according to Chalmers et al.^32^ counting all coding, somatic base substitutions and indels in the targeted regions, including synonymous alterations. “Variant calling” and “TMB calculation” were based on not related algorithms, thus the real number of coding variants cannot be derived from TMB and vice versa (see Manufacturer Instructions at https://emea.support.illumina.com/). The size of the targeted (coding) genomic region was 1.9 Mb. MSI as result of impaired DNA mismatch repair represents a phenotype of clinical significance in CRCs. A highly accurate exome-based predictive model for the MSI phenotype was used, it resides on a statistical MSI classifier from somatic mutation profiles that separates MSI-H (MSI-high) from MSS (MS stable) tumors^33^. The MSI classifier was trained using 999 exome-sequenced TCGA tumor samples with known MSI status (i.e. assayed from mononucleotide markers), and obtained a positive predictive value of 98.9% and a negative predictive value of 98.8% on an independent test set of 427 samples.

The set of somatic mutations observed in a cancer and distinct patterns of substitution types reflect the specific mutational processes that have been active during its life history. Here, with a descriptive aim, we reported the matched mutational profiles of primary and metastatic lesions displaying the fraction of mutations found in each trinucleotide context. A mutational signature is the combination of the frequencies of all base-pair mutation types (C:G>A:T, T:A>G:C, etc.) and their flanking nucleotides; the convention is to annotate mutations from the pyrimidine (C>A, T>A, etc.). The figures plot all 96 possible combinations of mutation types and neighboring bases.

### Tumor-infiltrating lymphocytes analysis

Analysis of T-cell subsets in tumor microenvironment was conducted through immunohistochemistry (IHC). Formalin-fixed, paraffin-embedded 4-μm tissue sections of primary CRC and lung metastases were immunostained according to a biotin-streptavidin-peroxidase method (YLEM kit, Rome, Italy). Before incubation with primary antibodies, sections were subjected to (i) routine deparaffinization, (ii) rehydration, (iii) treatment with Dako target retrieval solution, (iv) incubated for 10 min on a hot plate (95–99 °C), (v) allowed to cool for 20 min, (vi) incubated for 10 min in 3% hydrogen peroxide in distilled water, (vii) washed in PBS thrice for 5 min, (viii) incubated with 10% normal horse serum in PBS for 30 min, and (ix) washed with PBS buffer. Treatment with primary antibodies [anti-human CD3, anti-human CD8, anti-human FoxP3, anti-human Granzyme B^34^] was done for 2 h at room temperature. The antigens were then revealed through a biotin-labeled secondary antibody/streptavidin-peroxidase/diaminobenzidine tetrahydrochloride method: sections were incubated with biotin-labeled secondary antibody (1:30), streptavidin-peroxidase (1:30) for 20 min each, and stained for 5 min with 0.05% 3,3′-diaminobenzidine tetrahydrochloride freshly prepared in 0.05 mol/L Tris-HCl buffer (pH 7.6) containing 0.024% hydrogen peroxidase. Finally, slides were counterstained with hematoxylin, dehydrated, and mounted in Diatex. Negative controls were obtained by substituting the primary antibody with a mouse myeloma protein of the same subclass at the same concentration as the monoclonal antibody. Slides were scanned through an automated scanning microscope and image-analysis system (Genetix, San Jose, CA). Cell density was expressed as cells/mm^2^. All qualitative and quantitative analyses of T-cell subsets were reviewed by two pathologists (G.B., F.T.) blinded to all clinical information. The tumor microenvironment was morphologically divided into tumor core (TC) and invasive margins (IM) as previously described^11^.

### Bioinformatics analysis and data presentation

Illumina TruSigth Oncology 500 bioinformatics pipeline was applied to analyze sequencing results. A median of 118 million reads were generated for each sample and the coverage in the target region was above manufacturer’s suggested threshold of 150X. Sequence data were aligned to the human reference genome GRCh37 (http://www.ncbi.nlm.nih.gov/projects/genome/assembly/grc/human/index.shtml) using the Burrows–Wheeler Aligner with default parameters^35^. Both population- and cancer-specific variants were intersected with GENCODE, dbNSFP, ICGC-PCAWG, COSMIC, 1000Genomes, ClinVar, CancerMine, OncoScore, CIViC, CBMDB databases to assess the clinical significance of the found mutations. Variants were filtered with unmatched normal datasets and removed if the global minor allele frequency was <1%. The prioritization of variants was done according to a four-tiered structure, adopting the joint consensus recommendation by AMP/ACMG^36^. Variants of strong clinical significance in cancer were defined considering items with strongest evidence levels in the database for (i) clinical interpretations of variants in cancer (CIViC, civicdb.org) and (ii) Cancer Biomarkers (cancergenomeinterpreter.org/biomarkers). Results about variants shared between PTs and metastatic tumors (MTs) are shown with ID according to ClinVar; however, variants were also manually curated to exclude residual false positives. The scenario in which interpretation for the clinical consequence of a variant was “benign”, but the review status quality was scored <2/4 or reporting conflicting results, was defined as “probably benign”. The TP53 p.Pro72Arg variant was reported in this study as potentially relevant for CRC. In fact, while the codon 72 SNPs have limited impact on cancer risk for WT P53, many different research groups showed independently that this SNP markedly influences the activity of tumor-derived mutant forms of p53 (TP53) both in vitro and in vivo^37–41^. Complete sequences results can be accessed upon a signed justified request sent to genetica@centroames.it and ale.otto@libero.it.

The genetic sharing was indicated as the percent of mutational concordance in matched PTs and derivative MTs in all coding variations. Venn Diagrams were depicted in order to plot intersections among genetic results and Phenolyzer was used to evidence relevance and relationships between any “seed” genetic variants and “secondary” ones.

Phenolyzer is a computational tool that prioritize genes on the basis on updated existing knowledge (protein–protein interactions, sharing of biological pathways or gene family, gene–gene transcriptional regulation, etc.). It integrates OMIM, Orphanet, ClinVar, Gene Reviews and GWAS Catalog as gene-disease databases. However, for a complete methodology description see Yang et al.^42^ Results are expressed through a score system and a network visualization tool that integrates gene–gene and gene–cancer relationships providing readers with a panoramic view of the interactional context.

The disease-free interval (DFI) was measured in months and it represented the time elapsed from the surgical removal of PTs to the occurrence of lung metastases.

Densities of T-cell subsets were expressed as cells/mm^2^ and results represented with the arithmetic averages ± 2 standard deviations (SD). Cells were also manually counted by two Pathologists three times each. Box-and-wisher plots graphs with means ± 95% confidence intervals (CI) were also reported to provide an overview of results’ heterogeneity as well as to depict potential trends (means were compared with the *t*-test with Welch correction in case of unequal variances, *P* < 0.05 was considered statistically significant).

## Supplementary information

Supplementary figure legends

Supplementary table legend

Table S1

Supplementary data 2

Supplementary data 3

Supplementary data 4

Supplementary data 5

Supplementary data 6

Supplementary data 7

Supplementary data 8

Supplementary data 9

Supplementary data 10
