# Supplementary material for: Genetic trajectory and immune microenvironment of lung-specific oligometastatic colorectal cancer
Source: Cell Death Dis. 2020 Apr 24;11(4):275. doi: 10.1038/s41419-020-2480-6 (PMC7181838; doi:10.1038/s41419-020-2480-6)
Supplement: Supplementary file 1 — Supplementary figure legends [file 41419_2020_2480_MOESM1_ESM.docx]

**Supplementary figure legends**

**Data S2.**

Inclusion and exclusion criteria flow-chart for the studied cohort.

**Data S3.**

Mutational signatures of primary and metastatic tumors.

**Data S4.**

Flow-cytometric characterization of peripheral blood lymphocytes (rationale and methods).

**Data S5.**

Characterization of NK and T-regulatory cells.

**Data S6.**

Characterization of CD8+ cells.

**Data S7.**

Characterization of NK-mediated toxicity.

**Data S8.**

Supplementary references.

**Data S9.**

Hypothesis of different evolution trajectories among pluri-metastatic and oligo-metastatic CRC.

**Data S10.**

List of studied genes.
