# Supplementary material for: Genetic trajectory and immune microenvironment of lung-specific oligometastatic colorectal cancer
Source: Cell Death Dis. 2020 Apr 24;11(4):275. doi: 10.1038/s41419-020-2480-6 (PMC7181838; doi:10.1038/s41419-020-2480-6)
Supplement: Supplementary file 2 — Supplementary table legend [file 41419_2020_2480_MOESM2_ESM.docx]

**Supplementary table legend**

**Table S1.**

Results of studies reporting genetic evolution of matched primary and pluri-metastatic CRC.
