# Supplementary material for: Genetic trajectory and immune microenvironment of lung-specific oligometastatic colorectal cancer
Source: Cell Death Dis. 2020 Apr 24;11(4):275. doi: 10.1038/s41419-020-2480-6 (PMC7181838; doi:10.1038/s41419-020-2480-6)
Supplement: Supplementary file 3 — Table S1 [file 41419_2020_2480_MOESM3_ESM.docx]

| **Table S1.** Results of studies reporting genetic evolution of matched primary and pluri-metastatic CRC. **Supplementary data S1.** | | | | | | | | | | |
| --- | --- | --- | --- | --- | --- | --- | --- | --- | --- | --- |
| **Author** | **Year** | **No. of paired samples (PT/MT)** | **Patients’ characteristics at diagnosis** | **Site of metastases** | **NGS platform** | **Genetic sharing PT/MT (global concordance)** | **Four most frequent and shared mutations** | **Unshared altered genes in PT (found in primary only)** | **Unshared altered genes in MT**  **(found in metastasis only)** | **TMB** |
| Brannon AR  et al. | 2014 | 69 | Four pts stage II, 3 stage III, 62 stage IV. Seventy-five percent of metastases were synchronous. Thirty pts were chemonaive. | Liver (only two ovary). | Illumina, HiSeq 2000. | 79% | APC, TP53, KRAS, PI3KCA were the most frequently mutated genes and most highly concordant between PT and MT (concordance of KRAS, NRAS, or BRAF was 100%). | ALK, APC, ASXL1, BAP1, CARD11, CBL, CEBPA, EPHA3, EPHA6, EPHA7, EPHB1, ERBB2, ERBB4, FLT1, FOXL2, GRIN2A, KDM6A, KDR, LGR6, MDM4, MITF, NFKB2, NOTCH3, PBRM1, PDGFRB, PIK3CA, PIK3CD, PIK3CG, SMAD4, STK11, TET1, TP53, TSHR | APC, AR, ATM, ATRX, BCL6, BRCA2, EGFR, EPHA5, EPHA6, EPHB1, ERBB4, FAS, FH, FLT1, MAP2K1, MAP2K1, NF1, NFE2L2, NOTCH1, NTRK3, PIK3C2G, PIK3CA, PIK3CA, PIK3CD, PIK3CG, PIK3R1, PREX2, PTEN, PTPRS, REL, REL, SMAD4, SMAD4, SUFU, TBK1, TET1, TET2, TGFBR2, TP53, TSHR. | Not reported |
| Lee SY  et al. | 2014 | 15 | Stage IV. 6 pts had single liver metastasis. | Liver. | Illumina, HiSeq 2000. | *Mutational concordance showed only for each genes:  APC: 100%  TP53: 70%  KRAS: 100%  SMAD4: 75%. | APC, TP53, KRAS, SMAD4. APC and KRAS mutations were ever concordant between PT and MT. | BRAF, CTNNB1,  FBXW7, PIK3R1, TP53, SOX9. | ATR, BRAF, CDC42BPG, FBXW7, FLT4, KDR, PI3KCG, RB1, SMAD4, SOX9. | Not reported |
| Kim R  et al. | 2015 | 19 | Twelve pts were stage IV, 7 pts stage III. Data on treatments not reported. | Liver, lungs, lymphnodes, ovary. | Illumina, GAIIX. | 93.5% | APC and TP53 found concordant in 10/19 pairs.  KRAS ever concordant (9/19 pts). PI3K ever condordant (3/19 pts). | ABCA3, ADAMTS20, APC, BRCA2, CX3CR1, DGKB, ERBB4, HSP90AB1, ITGA10, ITGAL, JAK1, LRP1B, MACF1, MAP3K, MAGI2, MARK1, NTRK2, PARP14, PIK3CG, RASA1, ROBO1, SMAD2, SMAD3, SMAD4, TEX14, TNKS, TP53, TTN, WNT2, ZNF217, ZNF831. | ADAMTS18, ADAMTS20, ADCY1, APC, BCL9, CASC5, CHD5, CIC, COL7A1, CSMD3, EPHA5, ETV4, FANCG, FBXW7, GPC5, HERC1, KIAA1409, KNTC1, MACF1, MAPK10, MAST4, MGA, MGMTk, MMP2, MPL, MUC16, NOS1 , PCM1, PPM1H, PREX1, PRKCZ, PTPN13, PTPRC, PTPRD, RASA1, RB1CC1, ROBO1, RPS6KB2, SIRT6, SNX13, STIM1, TACR3, TCF12, TCF3, TOP2B, TOPBP1, TP53, TPO, TRAF4, TTN, VRTN, WNT2. | Not reported |
| Vignot S  et al. | 2015 | 13 | Stage IV. Six synchronous metastases, 7 methacronous. Patients received chemotherapy and/or radiotherapy (one pt) before surgery. | Liver and liver. Only local (1 pt), only peritoneum (1 pt). | Illumina, HiSeq 2000 | 78% | APC*,* TP53*,* KRAS*,* and SMAD4 were the most frequent mutated genes. Mutated APC had a concordance of 100%. | ALK, BRCA2, GNAS, NF1, RICTOR, STK11, TNKS | BRCA2, CDH2, CDKN2A, EPHB1, GLUCY1A2, PI3KCG, RB1, RET, SMO | Not reported |
| Kovaleva V  et al. | 2016 | 14 | Stage IV. Synchronous and/or metachronous liver and/or lung metastases. | Liver and lungs. | TruSeq Amplicon Cancer PanelTM, MiSeq (Illumina). | *From 0 to 100% (median 8.5%). | TP53, APC, KRAS, SMAD4. | ABL1, ATM, BRAF, EGFR, ERBB4, FBXW7, FGFR3, GNA11, GNAQ, HRAS, JAK3, KDR, KIT, MET, NOTCH1, NRAS, PDGFRA, PIK3CA, PTEN, RB1, RET, SMAD4, STK11, TP53, VHL | ABL1, AKT1, ALK, ATM, BRAF, CDH1, CDKN2A, CSF1R, CTNNB1, EGFR, ERBB2, ERBB4, FBXW7, FGFR2, FGFR3, FLT3, GNA11, GNAQ, GNAS, HNF1A, HRAS, IDH1, JAK3, KDR, KIT, KRAS, MET, MLH1, MPL, NOTCH1, NPM1, NRAS, PDGFRA, PIK3CA, PTEN, PTPN11, RB1, RET, SMAD4, SMARCB1, SMO, SRC, STK11, TP53, VHL. | Not reported |

*when the data were not clearly reported they were derived from Venn Diagrams or descriptive tables.
