# Supplementary figures and images for: Genetic trajectory and immune microenvironment of lung-specific oligometastatic colorectal cancer
Source: Cell Death Dis. 2020 Apr 24;11(4):275. doi: 10.1038/s41419-020-2480-6 (PMC7181838; doi:10.1038/s41419-020-2480-6)

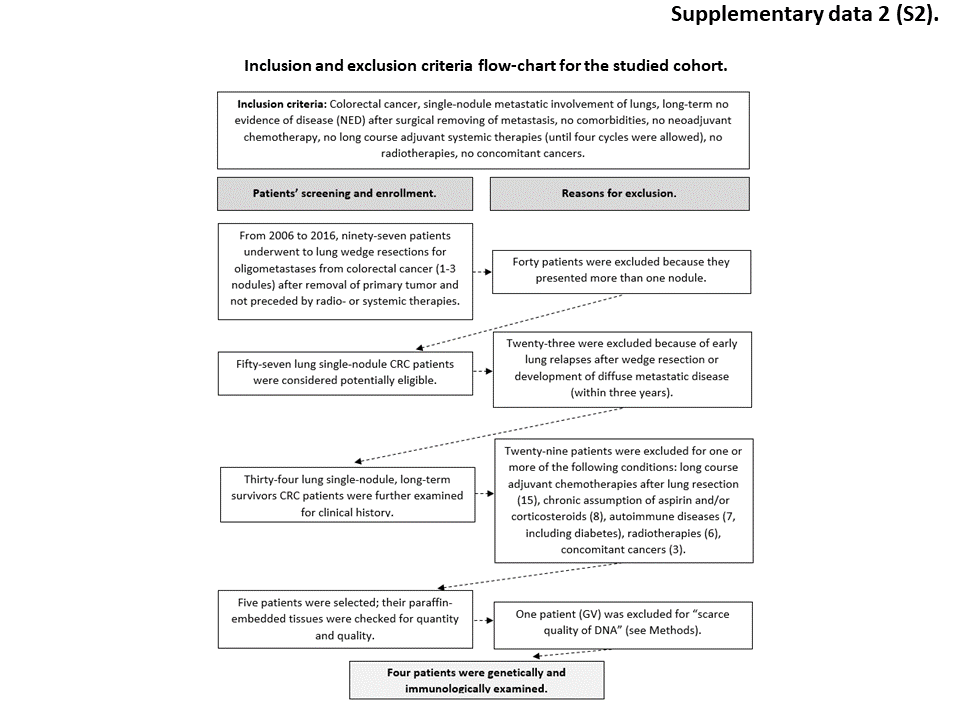

Supplement: Supplementary file 4 — Supplementary data 2 [file 41419_2020_2480_MOESM4_ESM.png]

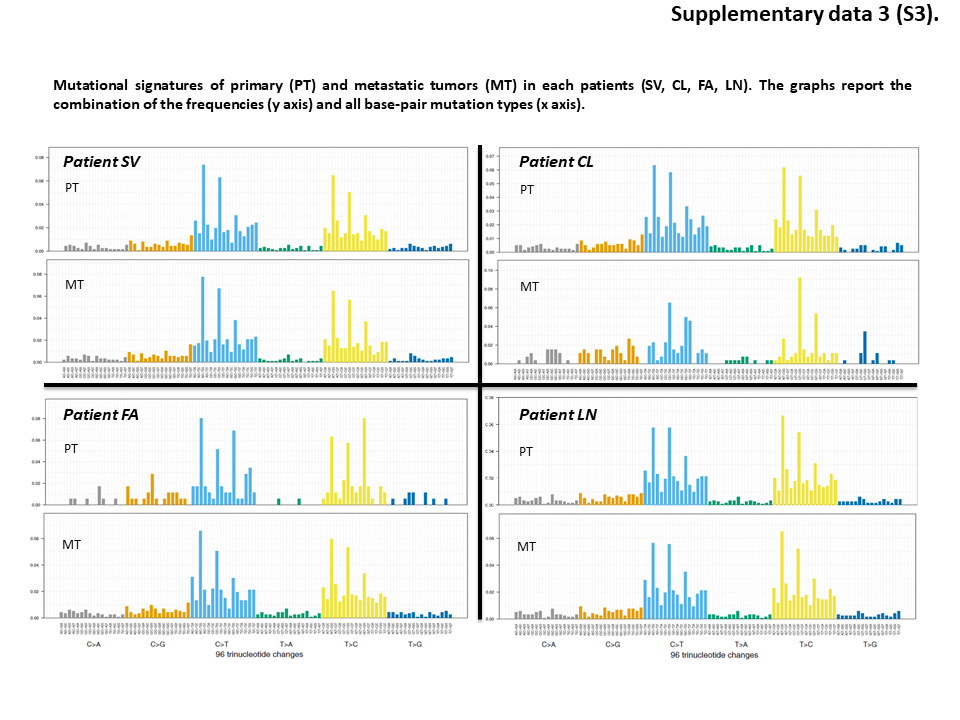

Supplement: Supplementary file 5 — Supplementary data 3 [file 41419_2020_2480_MOESM5_ESM.png]

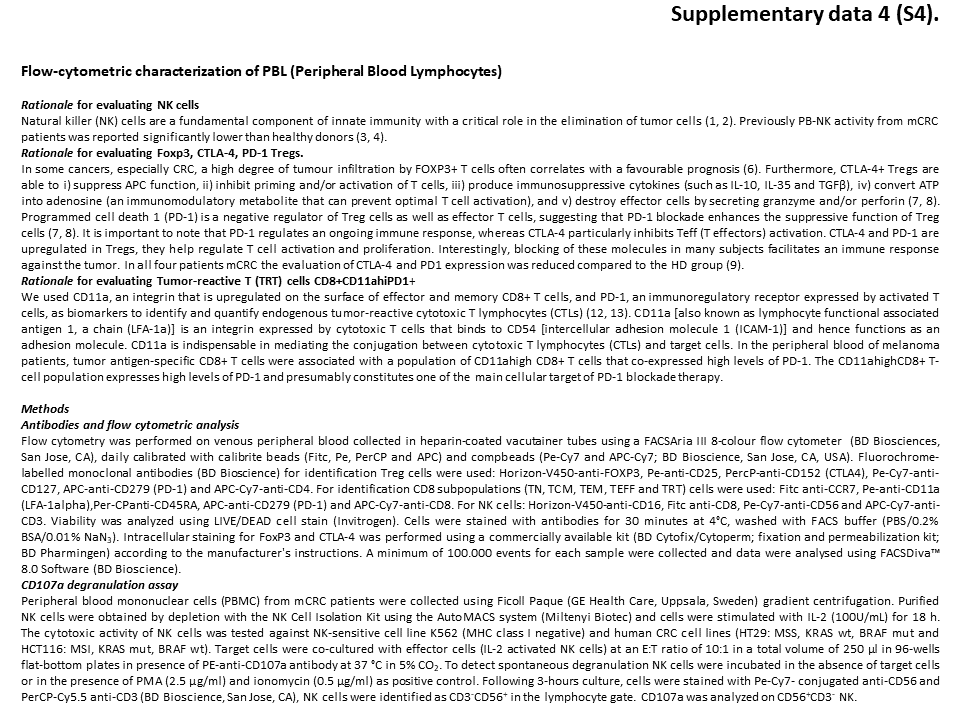

Supplement: Supplementary file 6 — Supplementary data 4 [file 41419_2020_2480_MOESM6_ESM.png]

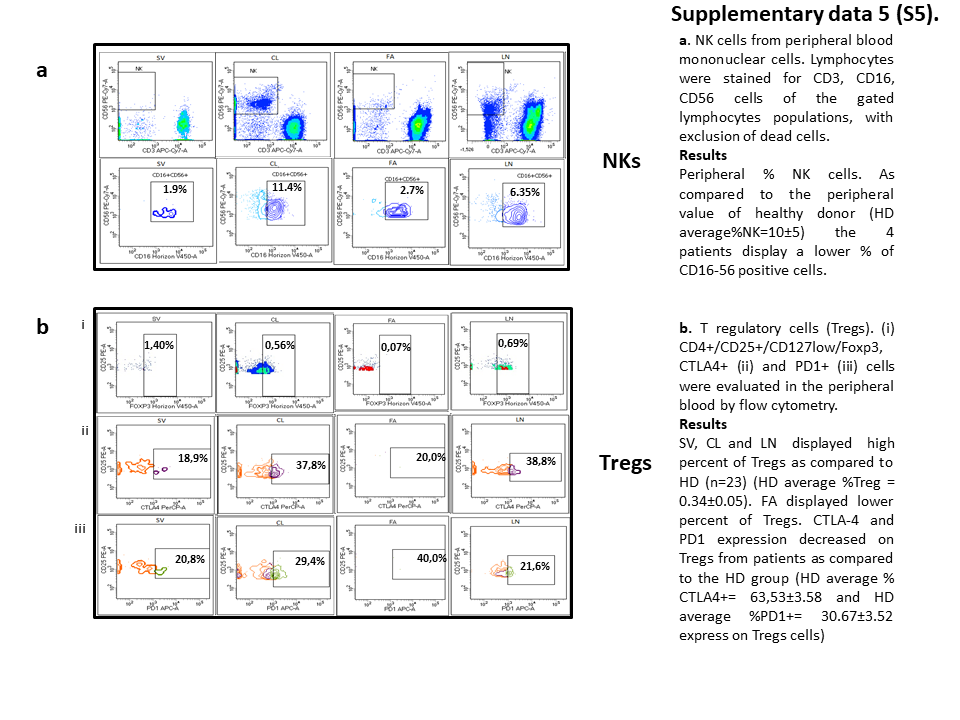

Supplement: Supplementary file 7 — Supplementary data 5 [file 41419_2020_2480_MOESM7_ESM.png]

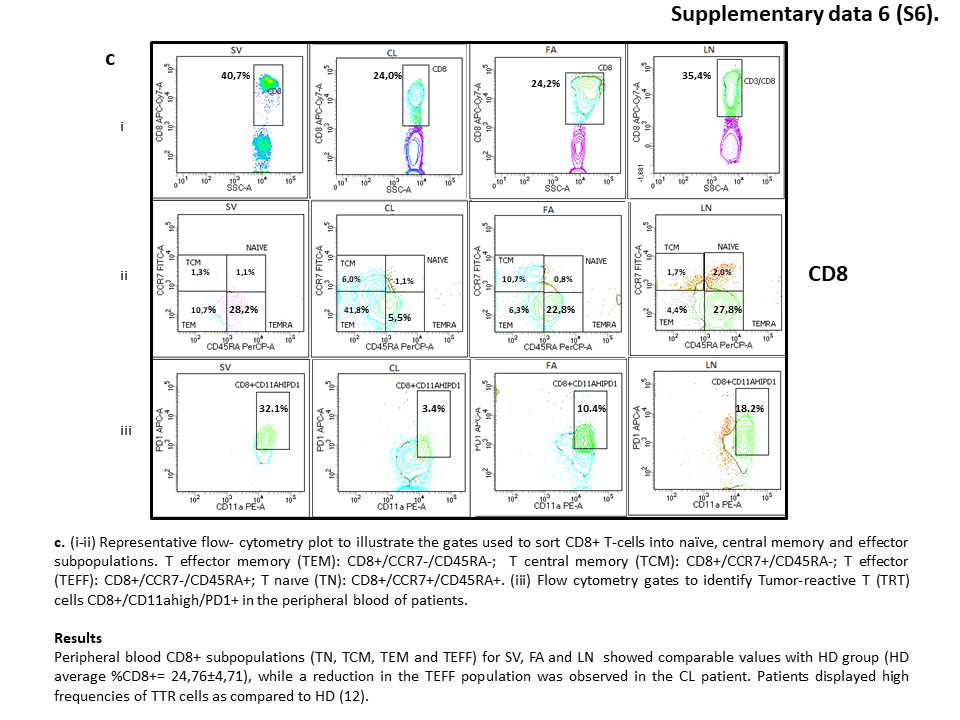

Supplement: Supplementary file 8 — Supplementary data 6 [file 41419_2020_2480_MOESM8_ESM.png]

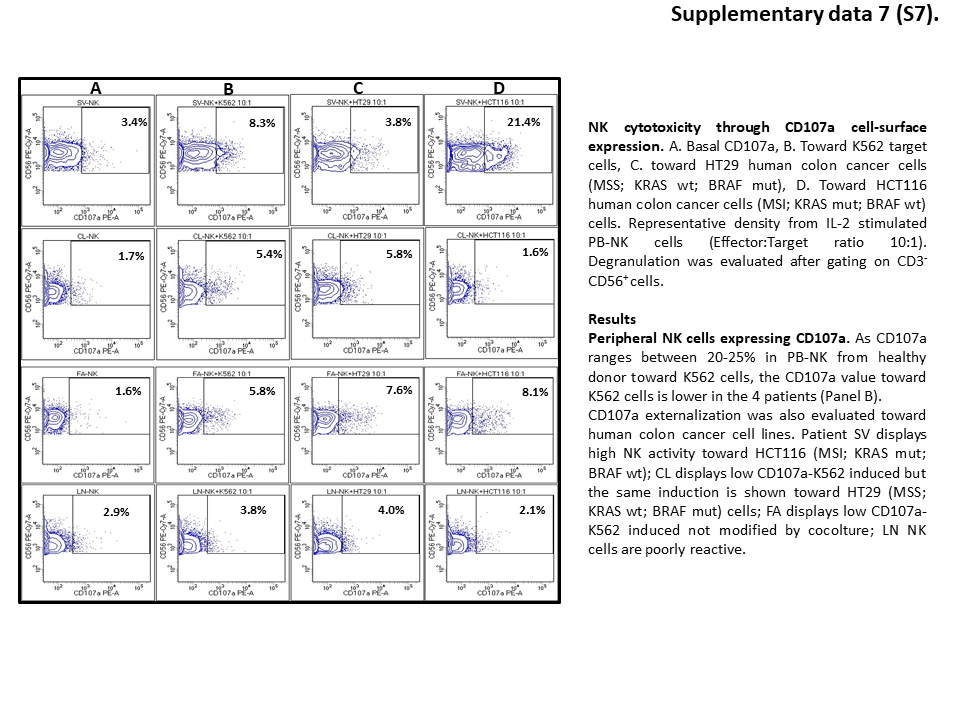

Supplement: Supplementary file 9 — Supplementary data 7 [file 41419_2020_2480_MOESM9_ESM.png]

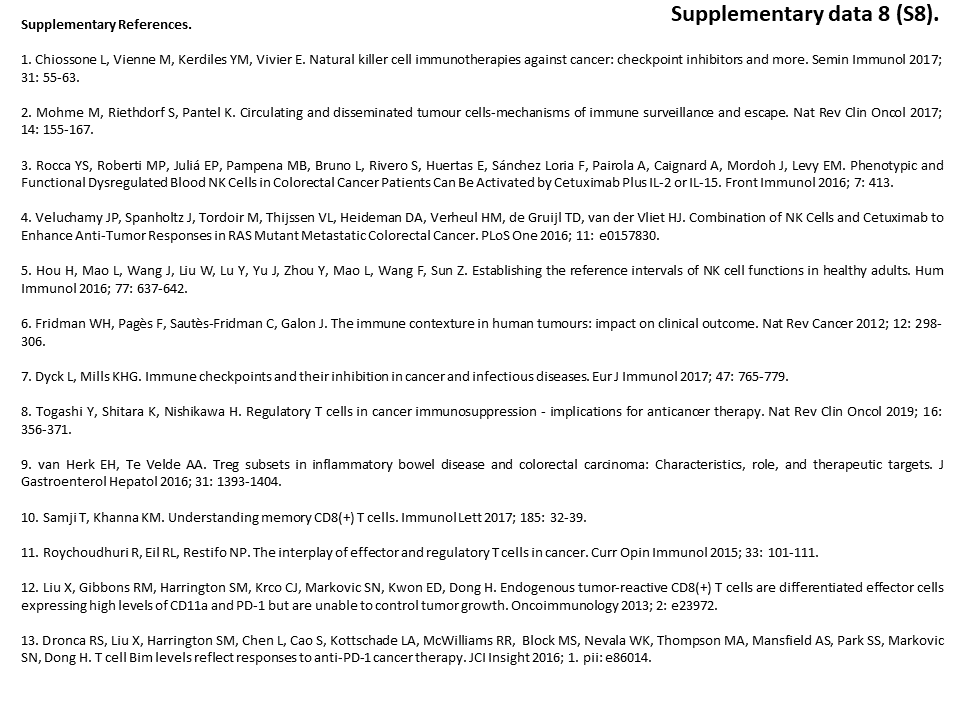

Supplement: Supplementary file 10 — Supplementary data 8 [file 41419_2020_2480_MOESM10_ESM.png]

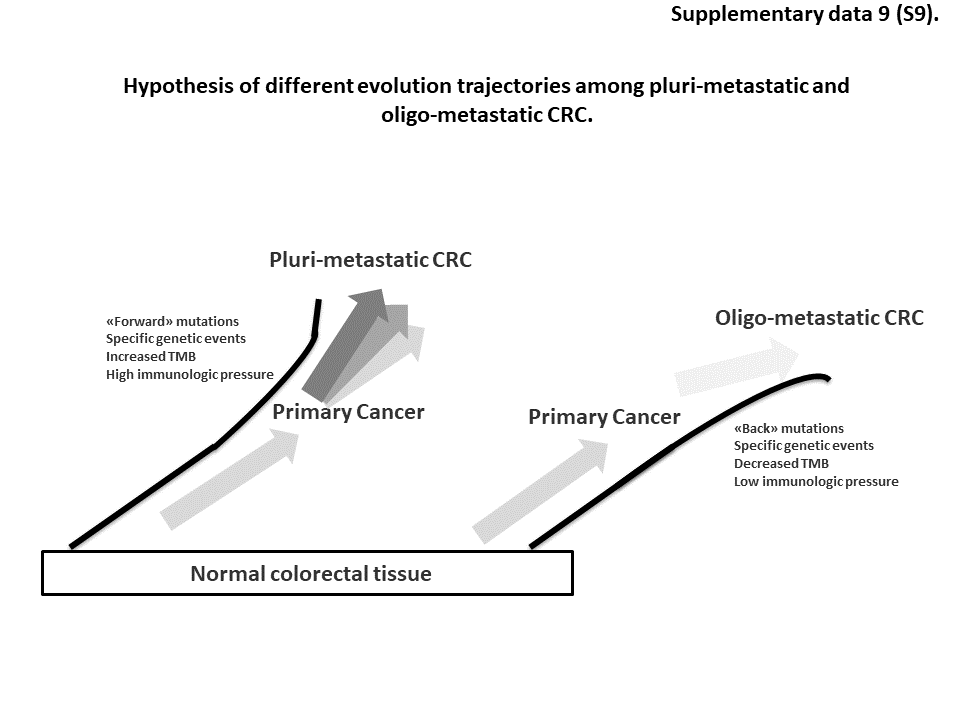

Supplement: Supplementary file 11 — Supplementary data 9 [file 41419_2020_2480_MOESM11_ESM.png]

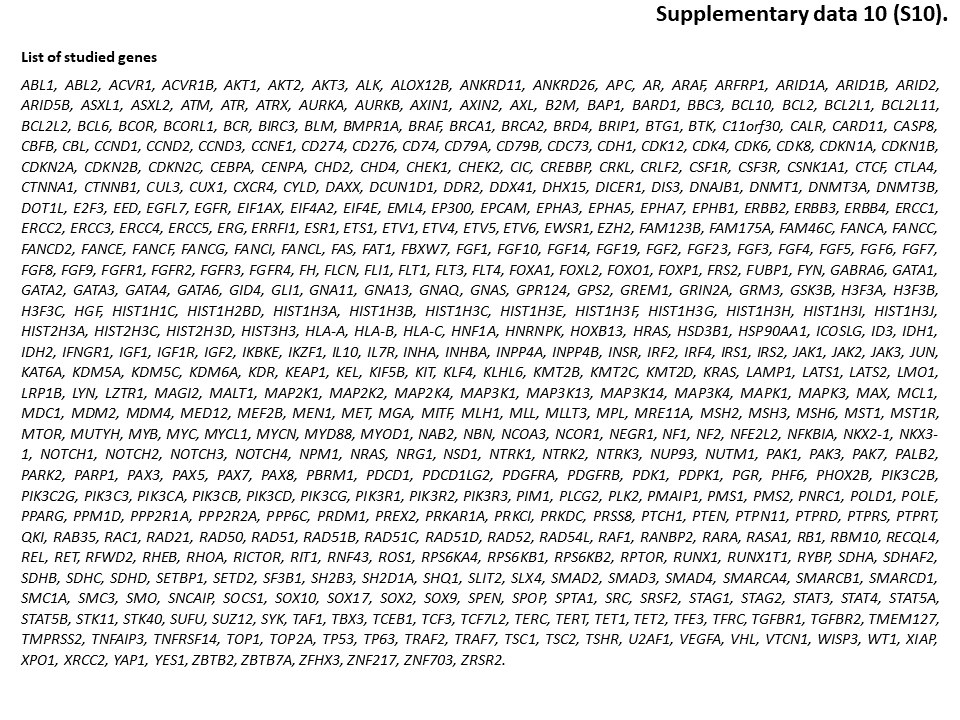

Supplement: Supplementary file 12 — Supplementary data 10 [file 41419_2020_2480_MOESM12_ESM.png]
